# Effect of Fat-Soluble Vitamins A, D, E and K on Vitamin Status and Metabolic Profile in Patients with Fat Malabsorption with and without Urolithiasis

**DOI:** 10.3390/nu12103110

**Published:** 2020-10-12

**Authors:** Roswitha Siener, Ihsan Machaka, Birgit Alteheld, Norman Bitterlich, Christine Metzner

**Affiliations:** 1 Department of Urology, University Stone Center, University Hospital Bonn, 53127 Bonn, Germany; ihsan.machaka@gmx.de; 2 Department of Nutrition and Food Sciences, Nutritional Physiology, University of Bonn, 53115 Bonn, Germany; b.alteheld@uni-bonn.de; 3 Department of Biostatistics, Medicine and Service Ltd., 09117 Chemnitz, Germany; bitterlich@medizinservice-sachsen.de; 4 Bonn Education Association for Dietetics r. A., 50935 Cologne, Germany; info-bfd@t-online.de or; 5 Clinic for Gastroenterology, Metabolic Disorders and Internal Intensive Medicine (Medical Clinic III), RWTH Aachen, 52074 Aachen, Germany

**Keywords:** intestinal resection, malabsorption, fat-soluble vitamins, vitamin D, vitamin E, ß-carotene, kidney stones, calcium oxalate stone formation, hyperoxaluria, hypocitraturia

## Abstract

Patients with intestinal fat malabsorption and urolithiasis are particularly at risk of acquiring fat-soluble vitamin deficiencies. The aim of the study was to evaluate the vitamin status and metabolic profile before and after the supplementation of fat-soluble vitamins A, D, E and K (ADEK) in 51 patients with fat malabsorption due to different intestinal diseases both with and without urolithiasis. Anthropometric, clinical, blood and 24-h urinary parameters and dietary intake were assessed at baseline and after ADEK supplementation for two weeks. At baseline, serum aspartate aminotransferase (AST) activity was higher in stone formers (SF; *n* = 10) than in non-stone formers (NSF; *n* = 41) but decreased significantly in SF patients after supplementation. Plasma vitamin D and E concentrations increased significantly and to a similar extent in both groups during intervention. While plasma vitamin D concentrations did not differ between the groups, vitamin E concentrations were significantly lower in the SF group than the NSF group before and after ADEK supplementation. Although vitamin D concentration increased significantly in both groups, urinary calcium excretion was not affected by ADEK supplementation. The decline in plasma AST activity in patients with urolithiasis might be attributed to the supplementation of ADEK. Patients with fat malabsorption may benefit from the supplementation of fat-soluble vitamins ADEK.

## 1. Introduction

The most common causes of fat malabsorption include the dysfunction of the intestinal epithelial barrier manifested by intestinal inflammation and disturbances in intestinal permeability [1,2,3,4] and exocrine pancreatic insufficiency [5]. Exocrine pancreatic insufficiency, associated with pancreatic diseases, such as chronic pancreatitis, pancreatic cancer, partial or total pancreatectomy and cystic fibrosis, and gastrointestinal disorders, including inflammatory bowel diseases, small bowel resections or bariatric surgical procedures, can result in maldigestion and malabsorption [5,6,7,8,9].

Urolithiasis is a frequent extraintestinal complication, particularly in patients undergoing ileal resection [10,11,12]. Whereas the prevalence of urinary stone disease in the general population has been reported to be between 4.7% and 8.8% [13,14], urolithiasis has been found to occur in 15.4% of patients with inflammatory bowel disease and up to 21% in patients with cystic fibrosis [10,15]. A recent population-based cohort study also reported a close association between chronic pancreatitis and urolithiasis [16].

Enteric hyperoxaluria is a major risk factor contributing to urinary stone formation in the setting of fat and bile acid malabsorption [17]. Unabsorbed fatty acids in the intestinal lumen bind to calcium, decreasing the ionized calcium concentration for complexation with oxalate [18]. With the depletion of free calcium, a larger percentage of unbound oxalate is available for absorption in the gut. In addition, it is postulated that unabsorbed fatty acids and bile salts increase colonic mucosa permeability for oxalate [19]. Previous studies have suggested that the degree of steatorrhea is correlated with urinary oxalate excretion in patients with fat malabsorption [18,20,21,22]. A deficiency in the oxalate-degrading bacteria *Oxalobacter formigenes* may also contribute to increased oxalate absorption [23].

Patients with intestinal fat malabsorption are particularly at risk of acquiring deficiencies in the fat-soluble vitamins A, D, E and K [24,25,26,27,28]. Clinical or subclinical deficiencies in fat-soluble vitamins may cause long-term health disorders, including neurological manifestations, osteoporosis and osteopenia, and result in higher morbidity and disease severity [5,25,29,30,31,32]. Thus far, studies on the vitamin status and the effect of the supplementation of fat-soluble vitamins in urinary stone formers with fat malabsorption are lacking. Therefore, the aim of this study was to evaluate vitamin status and metabolic profile before and after the oral supplementation of fat-soluble vitamins A, D, E and K (ADEK) in patients with fat malabsorption secondary to different intestinal diseases with and without a history of urolithiasis.

## 2. Materials and Methods

### 2.1. Study Design

A study in patients with fat malabsorption was conducted with the purpose of assessing the change in the fatty acid pattern of erythrocyte membrane phospholipids after the oral supplementation of specific fatty acids [33]. Data from the 2-week run-in period were evaluated for changes in the vitamin status and clinical and biochemical parameters before and after the administration of fat-soluble vitamins ADEK.

The inclusion criteria were fecal elastase-1 ≤ 200 μg/g of stool and/or clinical findings. Exclusion criteria were insulin-dependent diabetes accompanied by metabolic complications, renal replacement therapy, malignancies and pregnancy. The patients were instructed to discontinue fat-soluble vitamins other than the study supplements for at least four weeks prior to the run-in period until the end of the trial. All subjects received fat-soluble vitamins ADEK at a daily dose of 1.5 mg vitamin A (retinol equivalents), 9.6 μg vitamin D_3_, 198 mg vitamin E (tocopherol equivalents) and 0.09 mg vitamin K for 2 weeks [34].

Of 99 screened patients, 51 patients with diagnosed fat malabsorption due to different intestinal diseases were included into the prospective study. The cases consisted of 10 stone formers (SF) aged 37–69 years. Stone disease was confirmed by medical records (at least 1 documented event). Available stone analyses revealed calcium oxalate. According to medical history, fat malabsorption was present in patients before the diagnosis of stone disease. The controls were 41 non-stone formers (NSF), aged 20–76 years. Previous imaging findings showed no evidence of stone disease in patients in the NSF group.

Trained medical staff collected anthropometric data, dietary records and blood and 24-h urine samples before and two weeks after ADEK supplementation. Body weight was recorded on a digital scale, and height and waist circumference were measured using a height meter and measuring tape. During the study, patients consumed their self-selected diets. Dietary intake (3-day weighed food record) was calculated using PRODI, version 5.2 (Nutri-Science GmbH, Freiburg, Germany). The oxalate contents of the foods, which were measured at the laboratory of the University Stone Center of the University Hospital Bonn, were entered into the software database [35,36]. Venous blood samples were obtained after a nonsmoking and overnight fasting period of at least 12 h. Analysis of the clinical chemistry and biochemical blood parameters was performed using routine methods. The study was approved by the Ethics Committee of the Medical Faculty of the University of Bonn (07805) and informed consent was obtained from each patient before trial onset.

### 2.2. Analytical Procedures

Venous blood samples (using EDTA (ethylene diamine tetra acetic acid) tubes) were collected after an overnight fast and immediately centrifuged for the separation of plasma and blood cells (2800 × *g*, 10 min, 4 °C). The plasma was frozen in aliquots at −80 °C for a maximum of two months. The plasma samples were deproteinized by the addition of ethanol (containing apocarotinal as the internal standard, 5 μmol/L). Fat-soluble vitamins were extracted with n-hexane and analyzed using normal-phase high-performance liquid chromatography (column, Nucleosil 100-5 CN, 250 × 4.0 nm, Machery-Nagel, Düren, Germany) and ultraviolet detection (292 nm; coefficient of variation (CV) for retinol 4.3% and for tocopherol 4.1%). 25-Hydroxycholecalciferol was analyzed in plasma samples using an enzyme-linked immuno-sorbent assay (ELISA) kit (Immunodiagnosticsystems, Frankfurt, Germany) (CV 5.6%).

Total 8-isoprostane concentration (8-iso-prostaglandin F_2α_) was determined after alkaline hydrolysis of esterified isoprostanes and purification by solid phase extraction with a C-18 cartridge (Cayman Chemical, IBL, Hamburg, Germany) using an ELISA kit (Cayman Chemical) (CV 15%).

Feces were homogenized and a sample was preserved at −20 °C. Elastase-1 concentration was determined using an ELISA kit, with 2 monoclonal antibodies against 2 distinct specific epitopes of human pancreatic elastase-1 (Schebo-Biotec, Giessen, Germany) (CV 5.8%).

Urine volume, density, pH (potentiometry) and the concentrations of sodium, potassium and chloride (indirect ion selective electrode), calcium (cresolphthalein complex), magnesium (methylthymol blue), inorganic sulfate (nephelometry), inorganic phosphate (phosphate molybdate reaction), ammonium (ion selective electrode), creatinine (Jaffé reaction), citrate (enzymatically, citrate lyase), uric acid (enzymatically, uricase) and oxalate (enzymatically, oxalate oxidase) were measured.

### 2.3. Statistical Analysis

Statistical comparisons between groups were performed using the nonparametric Mann–Whitney U test. The nonparametric Wilcoxon matched-pairs signed-rank test was used for the comparison of changes at different time points within the study groups. The exact tests for small samples were used. Categorical variables were compared using Fisher’s exact test. The strength and direction of the linear relationship between 2 continuous variables before and after intervention, respectively, were expressed using Spearman’s rho correlation coefficient (R). Data are presented as mean ± standard deviation (SD) unless otherwise stated. All statistical tests were two-sided. Differences were considered statistically significant at *p* < 0.05. Statistical analysis was performed using SPSS^®^ for Windows, version 24.0 (IBM, Armonk, New York, NY, USA).

## 3. Results

### 3.1. Patients

Of 51 patients with fat malabsorption due to different intestinal diseases, ten patients had a history of urolithiasis. The characteristics and underlying intestinal disorders of the stone formers (SF) and non-stone formers (NSF) with fat malabsorption are presented in Table 1. The groups (SF and NSF) did not differ regarding gender distribution, age or anthropometric and cardiometabolic risk parameters. The rate of pancreatic and/or bowel resection was greater in the SF group compared to the NSF group (70% vs. 29%; *p* = 0.027). Pancreatic surgery with or without bowel resection was more prevalent in SF than NSF patients with exocrine pancreatic insufficiency (67% vs. 13%; *p* = 0.030). SF patients with Crohn’s disease and short bowel syndrome underwent extensive ileal resection and hemicolectomy, including the terminal ileum. 

### 3.2. Clinical Chemistry and Biochemical Characteristics

The total cholesterol and high-density lipoprotein (HDL) cholesterol concentrations were significantly lower in SF than in NSF patients before and after the oral supplementation of ADEK (Table 2). The low-density lipoprotein (LDL) cholesterol concentration was significantly lower in SF patients only after intervention. Serum aspartate aminotransferase (AST) activity was higher in SF than NSF patients at baseline but decreased significantly in SF patients after ADEK supplementation. A significant positive correlation between serum lipids triglycerides, total, HDL and LDL cholesterol and vitamin E concentration was found before ADEK (R = 0.333, *p* = 0.017; R = 0.621, *p* < 0.001; R = 0.376, *p* = 0.007; R = 0.467, *p* = 0.001; *n* = 51) and between triglycerides, total and LDL cholesterol and vitamin E concentration after ADEK supplementation (R = 0.413, *p* = 0.003; R = 0.615, *p* < 0.001; R = 0.519, *p* < 0.001; *n* = 51), respectively.

### 3.3. Vitamins D, E and Beta-Carotene

Mean plasma vitamin D concentration increased significantly and to a similar extent in both groups—i.e., by 5.72 nmol/L (12%) in the SF group and 5.66 nmol/L (11%) in the NSF group—after ADEK supplementation (Table 2). Vitamin D concentrations did not differ between both groups before and after intervention. Vitamin D deficiency, defined as a 25-hydroxyvitamin D (25(OH)D) concentration <50 nmol/L (<20 ng/mL) [37], was present in 61% of NSF patients and 70% of SF patients before, as compared to 54% and 60%, respectively, after intervention (Table 3). 

Mean plasma vitamin E concentration was significantly lower in SF than in NSF patients before and after the supplementation of ADEK. Vitamin E deficiency, defined as plasma vitamin E concentration <12 µmol/L [38], was present in 20% of SF patients but only in 2% of NSF patients at baseline as compared to 20% and 0% (*p* = 0.002), respectively, after intervention (Table 4). Plasma vitamin E concentrations increased significantly and to a similar extent in both groups—i.e., by 10.75 μmol/L (46% of baseline value) in the SF group and 17.26 μmol/L (58%) in the NSF group—after ADEK supplementation. 

Plasma beta-carotene concentration was almost twice as high in NSF patients than in SF patients before and after intervention (Table 2). Plasma beta-carotene concentration did not change after ADEK supplementation.

### 3.4. Urinary Parameters

Twenty-four-hour urinary oxalate was significantly higher and urinary citrate excretion was significantly lower in the SF group than the NSF group before and after ADEK supplementation, respectively (Table 5). No changes in urinary oxalate and citrate excretion were observed following ADEK supplementation. Twenty-four-hour urinary calcium excretion did not differ between the groups and did not change throughout the study. 

Urinary sulfate and magnesium excretion increased significantly in both groups during intervention. Urinary creatinine excretion was significantly higher in SF patients following ADEK supplementation but did not differ between SF and NSF patients before and after intervention. Urinary ammonium excretion increased significantly in SF patients during intervention and was significantly greater in the SF group than the NSF group after ADEK supplementation. 

A significant correlation was observed between urinary calcium excretion and plasma vitamin D concentration before (R = 0.283, *p* = 0.044) but not after ADEK supplementation (R = 0.183, *p* = 0.198; *n* = 51). Urinary citrate excretion was significantly and positively correlated with plasma beta-carotene concentration before (R = 0.557, *p* < 0.001) and after ADEK supplementation (R = 0.477, *p* < 0.001; *n* = 51), respectively. Urinary oxalate excretion was not consistently associated with beta-carotene concentration (R = −0.188, *p* = 0.188 before and R = −0.317, *p* = 0.023 after intervention, respectively; *n* = 51). 

### 3.5. Nutrient Intake

The mean dietary intakes of energy and the macronutrients protein, fat and carbohydrates were similar in SF and NSF patients before and after ADEK supplementation. No clinically relevant differences between both groups regarding other nutrients were observed during the study.

## 4. Discussion

Fat maldigestion and malabsorption is associated with increased risk for urolithiasis, particularly due to enteric hyperoxaluria and hypocitraturia. In patients with severe fat malabsorption, caused by different intestinal disorders, the resection status was found to be a major extrarenal risk factor for urinary stone formation [10,34,39]. In the present study, the rate of pancreatic and/or bowel resection and urinary oxalate excretion were significantly higher, while urinary citrate excretion was lower in the SF group than in the NSF group. The mechanisms potentially contributing to the intestinal hyperabsorption of dietary oxalate and secondary hyperoxaluria in SF patients include the malabsorption of fatty acids that bind calcium, leaving more free oxalate for absorption, and the increased concentration of fatty and bile acids causing increased colonic mucosa permeability [18,19,20]. The significantly lower serum lipids, not only total, but also HDL cholesterol, and higher serum AST activity in SF patients can be attributed to bile acid malabsorption and subsequent enhanced hepatic bile acid synthesis from cholesterol to compensate for severe bile acid losses [39,40]. Hypocitraturia can be ascribed to an insufficient secretion of sodium bicarbonate and bicarbonate losses in stool [7,34]. Although SF patients with severe intestinal fat malabsorption could be suspected to be more prone to developing deficiencies in the fat-soluble vitamins A, D, E and K than NSF patients, findings on the vitamin status and the effect of supplementation of fat-soluble vitamins in SF patients with fat malabsorption are lacking.

The lipophilic antioxidant vitamin E prevents unsaturated lipids in cell membranes from peroxidation by scavenging peroxyl radicals [38,41]. Damages caused by oxidative stress have been linked to numerous chronic disease conditions, including cancer and cardiovascular and neurodegenerative diseases [42]. The present study revealed that mean plasma vitamin E concentrations were significantly lower in the SF group than the NSF group before and after vitamin E supplementation. The supplementation of 198 mg (295 IU) of vitamin E (tocopherol equivalents) increased plasma vitamin E concentration significantly and to a similar extent in both groups. However, hypovitaminosis E, defined as plasma vitamin E concentration <12 µmol/L, was present in 20% of SF patients before and after ADEK supplementation and was significantly more prevalent in SF than NSF patients after intervention. Vitamin E deficiency occurred only in patients with pancreatic insufficiency, namely in two SF patients with resection and one NSF patient without resection. After intervention, 88% of NSF patients, but only 40% of SF patients, reached α-tocopherol concentrations of at least 30 µmol/L, the assumed optimal level of vitamin E for the prevention of nutrition-related diseases [38,43]. 

The present results of a high prevalence of vitamin E deficiency in SF patients with fat malabsorption secondary to exocrine pancreatic insufficiency are in accordance with findings from previous studies. A study in 40 chronic pancreatitis patients reported a vitamin E deficiency <16.5 µmol/L in 10% of patients [44]. In a prospective controlled cohort study of 62 chronic pancreatitis patients and 66 age-/sex-matched controls, 9.7% of patients were deficient in vitamin E, defined as serum vitamin E <21.3 µmol/L, compared to 1.5% of controls [25]. In a recent study in 301 chronic pancreatitis patients and 266 controls, 17.9% of patients, compared to 3.0% of controls, had α-tocopherol concentrations <5.7 mg/L [45]. Several studies have also documented significantly lower plasma α-tocopherol concentrations in patients with Crohn’s disease compared to controls [28,46,47,48]. 

Although both groups benefited from vitamin E supplementation, the prevalence of vitamin E deficiency remained unchanged and present only in SF patients. A retrospective chart review of patients with cystic fibrosis, and pancreatic insufficiency in 92%, revealed suboptimal serum vitamin E concentrations <12 µmol/L in 24% of patients at baseline [49]. Although the vitamin E supplement, on average, increased significantly by 12.2% annually from 218.38 to 351.58 IU (*p* < 0.0001), no parallel increase in serum α-tocopherol concentration was observed over the past five years, potentially due to ongoing issues with malabsorption. The findings from the present study support previous observations that plasma vitamin E concentration may be used as laboratory marker indicative of severe malabsorption and consequently malnutrition in patients with intestinal diseases and elevated risk of urinary stone formation [34,50]. 

Studies suggested that a decreased intraluminal concentration of bile salts is an important factor in the development of severe vitamin E deficiency [51,52]. In the present study, the significantly lower serum cholesterol concentrations in SF patients indicate compensatory hepatic bile acid synthesis from cholesterol [40]. Moreover, a significant positive correlation between vitamin E and serum lipids total cholesterol and triglycerides was found. The association of vitamin E with circulating lipid concentrations is well established and explained by the fact that vitamin E is transported in plasma in lipoproteins [53,54].

In addition to its property as provitamin A, the antioxidant beta-carotene is suggested to protect against lipid peroxidation in cell membranes and to enhance immune response [55,56]. In the present study, plasma beta-carotene concentrations were almost twice as high among NSF patients compared to SF patients at baseline and after two weeks, despite similar dietary intake. The mean beta-carotene concentrations of SF patients were constantly below 0.4 µmol/L, the assumed optimal plasma level of beta-carotene for the prevention of nutrition-related diseases [43]. Significantly lower plasma levels of beta-carotene were also observed in patients with Crohn’s disease compared to healthy controls [46,47]. Another study in Crohn’s disease patients revealed no differences in BMI, lipid peroxidation indexes or plasma antioxidant concentrations between patients with and without small-bowel resection, except for beta-carotene [57]. It is suggested that the antioxidant beta-carotene could be a marker for steatorrhea and, therefore, for the severity of fat malabsorption [34,58]. 

Vitamin D is essential for active intestinal calcium absorption and plays an important role in maintaining calcium homeostasis and bone health. Vitamin D is derived mainly from cutaneous synthesis in the presence of ultraviolet sunlight while dietary intake constitutes a minor fraction [37,59]. Low vitamin D status was found to be a biomarker for disease activity and a predictor of poor clinical outcomes in patients with inflammatory bowel disease [60,61]. Although vitamin D deficiency could be expected to be more frequent in SF patients with severe intestinal fat malabsorption, the prevalence of vitamin D concentrations below the common threshold of 50 nmol/L [37] was similar in SF and NSF patients before (70% vs. 61%, *p* = 0.725) and after ADEK supplementation (60% vs. 54%; *p* = 1.000). Moreover, the administration of 384 IU cholecalciferol resulted in a significant and similar increase in vitamin D concentrations in both groups. Plasma vitamin D concentrations did not differ between SF and NSF patients before (48.57 vs. 49.64; *p* = 0.788) and after ADEK supplementation (54.29 vs. 55.30; *p* = 0.752), which confirmed that plasma vitamin D concentrations in patients with fat malabsorption are rather associated with exposure to sunlight and not strongly influenced by vitamin D from diet or supplements [62]. A national health survey among adults in Germany reported serum 25(OH)D levels below 50 nmol/L in 61.6% of the participants and a mean serum 25(OH)D level of 45.6 nmol/L [63], which is comparable to the vitamin D status of our patients.

The findings of the present study are in accordance with the reported average vitamin D concentrations and the prevalence of vitamin D deficiency in patients with intestinal malabsorption syndromes, including chronic pancreatitis and inflammatory bowel diseases. A recent study in 301 chronic pancreatitis patients and 266 controls found similar vitamin D concentrations in patients and controls (20.7 vs. 22.2 ng/mL; *p* = 0.92 or 51.8 vs. 55.5 nmol/L) [45]. In a prospective controlled cohort study of 62 chronic pancreatitis patients and 66 age-/sex-matched controls, the reported prevalence of serum vitamin D deficiency (<50 nmol/L) did not significantly differ among patients and controls (58% vs. 61.7%; *p* = 0.894) [25]. In a retrospective study of 211 patients with inflammatory bowel diseases and 98 controls, vitamin D deficiency was present in 67.8% of patients and in 57.1% of controls [64].

Findings from studies on vitamin D status in urinary stone formers are conflicting. While a trial in 101 patients presenting with urolithiasis to a tertiary stone clinic found vitamin D deficiency only in 33.7% of patients [65], the prevalence of vitamin D deficiency <20 ng/mL was 56% in 884 patients with idiopathic calcium nephrolithiasis and 44% in 967 controls (*p* < 0.001), with median levels of 18 versus 23 ng/mL (*p* < 0.001) [66], and therefore was similar to the results of the present study. Meta-analysis of 32 observational studies involving 3510 stone formers and 19,718 controls showed that calcium stone formers and non-stone-formers had similar levels of vitamin D (25(OH)D) [67].

Vitamin D, as a key regulator of calcium and phosphate metabolism, is thought to play an important role in urinary stone formation. According to a systematic review and meta-analysis by Malihi et al. [68], long-term vitamin D supplementation resulted in increased risks of hypercalcemia and hypercalciuria, but not of kidney stones. Evidence from a systematic review and meta-analysis by Hu et al. [67] suggested that a higher level of 25-hydroxyvitamin D_3_ (25(OH)D), the major circulating metabolite of vitamin D, is significantly associated with hypercalciuric urolithiasis. While plasma vitamin D concentration increased significantly in SF and NSF patients in the present study, mean urinary calcium excretion was not affected by ADEK intervention. After ADEK supplementation, urinary calcium excretion exceeded the limit of hypercalciuria of 8 mmol/24 h in only one SF and two NSF patients (*p* = 1.000) compared to no SF and two NSF patients before intervention (*p* = 0.488). A possible explanation could be the low dose and the short period of vitamin D supplementation. On the contrary, vitamin D supplementation may have increased urinary magnesium excretion, an inhibitor of urinary stone formation, in the whole study group, without affecting serum magnesium concentration. 

Hyperoxaluria and hypocitraturia, the major urinary risk factors for stone formation in patients with fat malabsorption, were significantly more prevalent in SF than NSF patients and did not change during ADEK supplementation. Urinary citrate excretion was significantly and positively correlated with plasma beta-carotene concentration before and after supplementation, while a significant inverse relationship between urinary oxalate excretion and plasma beta-carotene concentration was observed only after ADEK supplementation. Given that urinary stone formation is associated with high oxidative stress and damage to renal tubular cells in humans, it is suggested that higher beta-carotene status could decrease the risk of urinary stone formation [69,70]. A study in 17,695 participants in the National Health and Nutrition Examination Survey (NHANES III) demonstrated a significant inverse association between serum beta-carotene concentrations and the prevalence of urinary stone disease [70]. The present results support the findings that beta-carotene may play a role in urinary stone formation. 

## 5. Conclusions

This is the first study to evaluate the effect of the supplementation of fat-soluble vitamins A, D, E and K on vitamin status and the metabolic profile of urinary stone formers with intestinal fat malabsorption. The supplementation of ADEK resulted in beneficial changes in serum AST activity in SF patients and plasma vitamin E and D concentrations in both groups. The significantly higher serum AST activity and lower total cholesterol, HDL cholesterol, beta-carotene and vitamin E concentrations before and after supplementation suggest that SF patients suffer from more severe malabsorption than NSF patients. The findings from the present study support previous observations that plasma vitamin E and beta-carotene concentrations may be used as laboratory markers to indicate severe malabsorption in patients with intestinal diseases and elevated risk of urinary stone formation. Urinary magnesium excretion, an inhibitor of stone formation, increased significantly in both groups after intervention. Plasma beta-carotene concentration was positively correlated with urinary citrate and was inversely related to urinary oxalate excretion after ADEK supplementation. These results suggest that higher beta-carotene status could decrease the risk of urinary stone formation. Patients with fat malabsorption may benefit from the supplementation of fat-soluble vitamins ADEK and beta-carotene due to enhanced antioxidant capacities. Further research is needed to evaluate the impact of each fat-soluble vitamin and beta-carotene on metabolic profile and risk factors for stone formation in SF patients with fat malabsorption secondary to intestinal diseases.

## Figures and Tables

**Table 1:** Baseline patient characteristics.

	Stone Formers	Non-Stone Formers	*p* Value
Patients (*n*)	10	41	
• Men (*n*)	6 (60%)	12 (29%)	0.138
• Women (*n*)	4 (40%)	29 (71%)	
Age (years)	56.1 ± 12.6	48.3 ± 14.2	0.136
Height (cm) ^1^	171.3 ± 5.9	170.5 ± 9.6	0.260
Weight (kg) ^1^	74.9 ± 15.0	70.4 ± 12.4	0.454
Body mass index (kg/m^2^) ^1^	25.4 ± 4.3	24.3 ± 4.5	0.436
Waist circumference (cm; men) ^1^	100 ± 11	92 ± 9	0.219
Waist circumference (cm; women)	79 ± 5	83 ± 11	0.611
Systolic blood pressure (mm Hg)	129 ± 17	125 ± 25	0.337
Diastolic blood pressure (mm Hg)	86 ± 12	83 ± 12	0.469
Resting heart rate (1/min)	70 ± 11	68 ± 9	0.974
Smokers (*n*)	3 (30%)	11 (27%)	1.000
Confirmed diagnosis	10	41	0.298
Exocrine pancreatic insufficiency	6	15	
• Previous pancreatic surgery	4 ^a^	2 ^b^	
Crohn’s disease	2	13	
• Small bowel resection	0	3	
• Colon resection	0	1	
• Small bowel and colon resection	2	6	
Cystic fibrosis	0	2	
Celiac disease	0	5	
Primary biliary cirrhosis	1	1	
Liver cirrhosis	0	1	
Short bowel syndrome	1	0	
Idiopathic malabsorption	0	4	

^1^ Total of 50 because of missing value (wheelchair driver). ^a^ Of four procedures, one was Whipple resection, one was pylorus-preserving Whipple procedure and hemicolectomy due to necrosis and two were partial pancreatic resections. ^b^ Of two procedures, one was partial pancreatic resection and one was pancreatic tail resection and pancreatic jejunostomy. Data are presented as mean ± standard deviation or number; *p* Value: Mann–Whitney U test or Fisher’s exact test.

**Table 2:** Clinical chemistry and biochemical characteristics of stone formers and non-stone formers at baseline and after ADEK supplementation.

	SF (*n* = 10)	NSF (*n* = 41)	Total (*n* = 51)		SF (*n* = 10)	NSF (*n* = 41)	Total (*n* = 51)			
	Baseline	Baseline	Baseline		Week 2	Week 2	Week 2			
	Mean ± SD	Mean ± SD	Mean ± SD	*p* Value ^a^	Mean ± SD	Mean ± SD	Mean ± SD	*p* Value ^a^	*p* Value ^b^	*p* Value ^c^
Total protein (g/L)	72.1 ± 4.7	72.2 ± 5.1	72.1 ± 4.9	0.958	72.3 ± 4.5	72.6 ± 6.4	72.6 ± 6.1	0.703	0.793	0.968
Albumin (g/L) ^1^	42.5 ± 4.7	43.0 ± 4.4	42.9 ± 4.4	0.766	43.3 ± 5.7	43.0 ± 4.7	43.0 ± 4.9	0.689	0.994	0.392
Creatinine (mg/dL)	0.93 ± 0.29	0.81 ± 0.19	0.83 ± 0.22	0.223	0.87 ± 0.29	0.82 ± 0.26	0.83 ± 0.27	0.784	0.341	0.109
Total cholesterol (mg/dL)	169 ± 53	206 ± 50	199 ± 52	0.036	166 ± 57	209 ± 53	200 ± 56	0.022	0.998	0.129
HDL cholesterol (mg/dL)	43 ± 9	56 ± 15	54 ± 15	0.003	42 ± 11	56 ± 16	54 ± 16	0.004	0.532	0.686
LDL cholesterol (mg/dL)	103 ± 45	127 ± 44	123 ± 45	0.072	100 ± 45	130 ± 46	124 ± 47	0.041	0.666	0.363
Triglycerides (mg/dL)	129 ± 75	129 ± 114	129 ± 106	0.704	111 ± 51	120 ± 79	118 ± 74	0.967	0.551	0.237
AST (U/L)	34 ± 14	30 ± 45	31 ± 40	0.005	30 ± 11	29 ± 40	29 ± 36	0.022	0.074	0.014
GGT (U/L)	71 ± 113	36 ± 33	43 ± 58	0.967	62 ± 85	37 ± 39	42 ± 51	0.893	0.756	0.643
Uric acid (mg/dL)	4.8 ± 0.9	4.7 ± 1.2	4.7 ± 1.1	0.856	4.5 ± 1.1	4.8 ± 1.2	4.7 ± 1.2	0.627	0.294	0.308
8-Isoprostanes (pmol/L) ^1^	28.40 ± 17.39	28.79 ± 28.67	28.71 ± 26.64	0.624	23.24 ± 13.48	27.15 ± 25.99	26.38 ± 23.99	0.991	0.249	0.436
Magnesium (mmol/L) ^1^	0.75 ± 0.11	0.79 ± 0.07	0.78 ± 0.08	0.369	0.75 ± 0.09	0.80 ± 0.08	0.79 ± 0.09	0.053	0.890	0.628
Homocysteine (µmol/L)	10.85 ± 3.98	11.46 ± 5.25	11.34 ± 4.99	0.986	11.24 ± 4.75	11.62 ± 5.17	11.55 ± 5.05	0.876	0.617	0.524
Folic acid (ng/mL]	8.28 ± 4.62	7.44 ± 5.22	7.61 ± 5.07	0.344	8.27 ± 4.19	7.15 ± 4.20	7.37 ± 4.18	0.307	0.950	0.949
Beta-carotene (µmol/L)	0.27 ± 0.45	0.49 ± 0.41	0.45 ± 0.42	0.006	0.28 ± 0.42	0.49 ± 0.41	0.44 ± 0.41	0.011	0.632	0.752
Vitamin A (µmol/L)	1.39 ± 0.85	1.55 ± 0.61	1.52 ± 0.66	0.447	1.36 ± 0.82	1.50 ± 0.61	1.47 ± 0.65	0.392	0.101	0.599
Vitamin D (nmol/L)	48.57 ± 31.49	49.64 ± 23.93	49.43 ± 25.24	0.788	54.29 ± 32.59	55.30 ± 22.24	55.10 ± 24.23	0.752	<0.001	0.752
Vitamin E (µmol/L)	23.30 ± 17.88	29.54 ± 9.77	28.31 ± 11.84	0.039	34.05 ± 25.67	46.80 ± 15.69	44.30 ± 18.48	0.036	<0.001	0.168

Abbreviations: ADEK, vitamins A, D, E and K; AST, aspartate aminotransferase; GGT, gamma-glutamyl transferase; HDL, high-density lipoprotein; LDL, low-density lipoprotein; NSF, non-stone formers; SD, standard deviation; SF, stone formers; ^1^ Total of 50 because of missing value; ^a^ Mann–Whitney U test; *p* values for comparison between SF and NSF; ^b^ Wilcoxon signed-rank test; *p* values for comparison between week 2 and baseline; ^c^ Mann–Whitney U test; *p* values for comparison of group × time interactions.

**Table 3:** Plasma vitamin D status of stone formers and non-stone formers at baseline and after ADEK supplementation.

	Vitamin D	SF (*n* = 10)	NSF (*n* = 41)	Total (*n* = 51)	*p* Value
	nmol/L	*n* (%)	*n* (%)	*n* (%)	
Baseline	<50	7 (70%)	25 (61%)	32 (63%)	0.725
	≥50	3 (30%)	16 (39%)	19 (37%)	
Week 2	<50	6 (60%)	22 (54%)	28 (55%)	1.000
	≥50	4 (40%)	19 (46%)	23 (45%)	

Abbreviations: NSF, non-stone formers; SF, stone formers; Fisher’s exact test; *p* value for comparison between SF and NSF.

**Table 4:** Plasma vitamin E status of stone formers and non-stone formers at baseline and after ADEK supplementation.

	Vitamin E	SF (*n* = 10)	NSF (*n* = 41)	Total (*n* = 51)	*p* Value
	µmol/L	*n* (%)	*n* (%)	*n* (%)	
Baseline	<12	2 (20%)	1 (2%)	3 (6%)	0.132
	12–29	5 (50%)	22 (54%)	27 (53%)	
	≥30	3 (30%)	18 (44%)	21 (41%)	
Week 2	<12	2 (20%)	0 (0%)	2 (4%)	0.002
	12–29	4 (40%)	5 (12%)	9 (18%)	
	≥30	4 (40%)	36 (88%)	40 (78%)	

Abbreviations: NSF, non-stone formers; SF, stone formers; Fisher’s exact test; *p* value for comparison between SF and NSF.

**Table 5:** Urinary parameters of stone formers and non-stone formers at baseline and after ADEK supplementation.

	SF (*n* = 10)	NSF (*n* = 41)	Total (*n* = 51)		SF (*n* = 10)	NSF (*n* = 41)	Total (*n* = 51)			
	Baseline	Baseline	Baseline		Week 2	Week 2	Week 2			
	Mean ± SD	Mean ± SD	Mean ± SD	*p* Value ^a^	Mean ± SD	Mean ± SD	Mean ± SD	*p* Value ^a^	*p* Value ^b^	*p* Value ^c^
Volume (L/24 h)	2.296 ± 0.889	2.142 ± 0.765	2.172 ± 0.784	0.472	2.060 ± 1.107	2.220 ± 0.914	2.189 ± 0.945	0.479	0.951	0.201
Density (g/cm^3^)	1.008 ± 0.005	1.008 ± 0.005	1.008 ± 0.005	0.747	1.010 ± 0.006	1.008 ± 0.005	1.008 ± 0.005	0.275	0.396	0.195
pH	6.01 ± 0.67	6.04 ± 0.48	6.03 ± 0.52	0.775	5.91 ± 0.62	6.10 ± 0.51	6.06 ± 0.53	0.299	0.634	0.193
Sodium (mmol/24 h)	168 ± 66	146 ± 67	150 ± 67	0.253	185 ± 60	150 ± 63	157 ± 63	0.127	0.477	0.717
Potassium (mmol/24 h)	51 ± 22	58 ± 18	56 ± 19	0.330	51 ± 21	56 ± 21	55 ± 21	0.586	0.869	0.803
Calcium (mmol/24 h)	3.55 ± 2.33	3.61 ± 1.99	3.60 ± 2.04	0.991	4.15 ± 2.54	3.84 ± 2.11	3.90 ± 2.18	0.735	0.064	0.379
Magnesium (mmol/24 h)	2.84 ± 1.90	3.51 ± 1.78	3.38 ± 1.80	0.392	3.31 ± 2.29	3.81 ± 1.39	3.71 ± 1.59	0.379	0.003	0.583
Ammonium (mmol/24 h)	25.4 ± 16.6	23.6 ± 11.6	23.9 ± 12.5	0.895	31.4 ± 17.3	23.8 ± 15.5	25.3 ± 16.0	0.049	0.294	0.010
Chloride (mmol/24 h)	199 ± 74	179 ± 67	183 ± 68	0.307	220 ± 64	178 ± 65	186 ± 66	0.069	0.967	0.319
Phosphate (mmol/24 h)	26.8 ± 13.1	25.1 ± 7.5	25.4 ± 8.7	0.824	31.5 ± 16.2	26.0 ± 8.6	27.1 ± 10.5	0.461	0.223	0.140
Sulfate (mmol/24 h)	16.8 ± 7.6	17.1 ± 5.6	17.0 ± 6.0	0.752	19.5 ± 5.6	18.5 ± 6.5	18.7 ± 6.3	0.843	0.017	0.330
Creatinine (mmol/24 h)	9.76 ± 3.69	10.35 ± 2.74	10.23 ± 2.92	0.379	12.11 ± 4.54	10.46 ± 3.66	10.78 ± 3.86	0.354	0.249	0.008
Uric acid (mmol/24 h)	2.59 ± 1.14	3.17 ± 0.82	3.06 ± 0.91	0.175	3.14 ± 1.04	3.23 ± 0.82	3.21 ± 0.85	0.735	0.273	0.105
Oxalate (mmol/24 h)	0.649 ± 0.442	0.395 ± 0.215	0.445 ± 0.287	0.049	0.659 ± 0.292	0.378 ± 0.168	0.433 ± 0.225	0.002	0.900	0.168
Citrate (mmol/24 h)	1.630 ± 1.645	2.933 ± 1.848	2.678 ± 1.868	0.039	1.606 ± 1.824	3.156 ± 1.968	2.852 ± 2.021	0.027	0.091	0.199

Abbreviations: NSF, non-stone formers; SD, standard deviation; SF, stone formers; ^a^ Mann–Whitney U test; *p* values for comparison between SF and NSF; ^b^ Wilcoxon signed-rank test; *p* values for comparison between week 2 and baseline; ^c^ Mann–Whitney U test; *p* values for comparison of group × time interactions.
